# In vitro digestibility and ruminal methane production of the predominant Namibian rangeland encroacher bush species collected in two different seasons

**DOI:** 10.1007/s11250-026-05093-8

**Published:** 2026-06-01

**Authors:** Shiningavamwe Katrina Lugambo, Mupangwa Johnfisher, Lutaaya Emmanuel, Halmemies-Beauchet-Filleau Anni, Vanhatalo Aila

**Affiliations:** 1 Ministry of Agriculture, Water and Land Reform, Windhoek, Namibia; 2 Department of Animal Production, Agribusiness and Economics, University of Namibia, Windhoek, Namibia; 3 Department of Agricultural Sciences, University of Helsinki, Helsinki, Finland

**Keywords:** Browse, Indigestible NDF, Bush encroachment, Climate change adaptation, Greenhouse gases

## Abstract

This study evaluated the in situ neutral detergent fibre (NDF) digestibility, in vitro organic matter digestibility (OMD) and methane production of the encroacher species *Senegalia mellifera*,* Dichrostachys cinerea*,* Terminalia sericea* and *Rhigozum trichotomum*. Leaves and twigs (≤ 20 mm) were harvested during late dry and early rainy seasons. The indigestible neutral detergent fibre (iNDF) and digestible neutral detergent fibre (dNDF) were determined by the *in sacco* nylon bag technique. The in vitro organic matter digestibility (OMD) of bush samples were based on the two-stage pepsin + cellulase solubility technique. Methane gas production was determined by in vitro method. The iNDF for *D*. *cinerea* and *S*. *mellifera* decreased (*P* < 0.0001) by 4% from late dry to early rainy season, while that of *R. trichotomum* increased by 13% and *T*. *sericea* increased by 3%. Digestible NDF decreased (*P* < 0.001) by 7% − 29% from late dry to early rainy season for all species, except *S*. *mellifera*. Species and species x season interaction affected (*P* < 0.0001) OMD. Except for *S. mellifera*, the in vitro OMD decreased (*P* < 0.001) from late dry to early rainy season. In vitro methane gas production of all species was higher (*P* = 0.0004) by 18% − 58% during the late dry season compared to the early rainy season (147.6 versus 92.0 mL/g DM). In conclusion, based on the low OMD and high iNDF, the studied species may require further intervention to improve their digestibility and feeding value.

## Introduction

Worldwide rangelands support the livelihoods of over a billion people through livestock farming (Millenium Ecosystem Assessment 2005). Land degradation through extensive bush encroachment in arid and semiarid areas is a worldwide problem that threatens food security and livelihoods and is exacerbated by climate change (O’Connor et al. 2014). Bush encroachment threatens sustainability and profitability of cattle farming (Espach 2006; Borner et al. 2007). In Namibia, livestock production is a major economic and livelihood activity, which contributes at least 75% to total agricultural output, with beef production being in the lead, followed by sheep and goats production (MAWF 2012). However, the industry faces challenges, amongst which bush encroachment and prolonged droughts pose major threats (Oosthuizen and Laubscher 2019).

Although browse plants, in general, constitute an important feed resource for ruminants, the encroaching nature of woody species such as *Senegalia mellifera*, *Vachellia karoo* and *Terminalia* species, makes rangeland habitats inaccessible to livestock and game animals. In addition, bush encroachment causes reduction of the rangeland carrying capacity, livestock productivity (Kabeta et al. 2020; Marquart et al. 2023) and hence economic losses to the livestock industry and agricultural sector in general. In Namibia, the economic impact of bush encroachment to the agricultural sector is estimated to be more than one billion Namibian dollars (MAWF 2012; Birch et al. 2016; Shikangala and Mapani 2020). Browse leaves, twigs and pods provide a considerable amount of biomass for livestock especially during the dry season when other feed resources are depleted (Moleele 1998; Kazemi and Bezdi 2021; Kearns et al. 2023). Hence, interventions are needed to convert massive encroacher bush biomass resources into livestock fodder.

In recent years, research has focused its attention on the utilization of encroacher bushes as animal feed as a climate change adaptation strategy (Mupangwa et al. 2023). Widespread adoption of feeding ruminants on bush-based rations from encroacher bushes has been gaining momentum in Namibia (Honsbein et al. 2018; Mupangwa et al. 2023), both as a drought relief strategy and also for commercial purposes. There is still, however, a need for an adequate understanding of the feeding potential of encroacher species when harvested as milled bush, in order to ensure their appropriate use in ruminant nutrition. In an era of climate change, the woody vegetation will be a major contributor to animal feed both livestock and game in many countries the world over, as the rangeland herbaceous vegetation fail to grow due to limited rainfall.

Milled bush is mainly composed of the structural carbohydrate neutral detergent fibre (NDF), which predominantly consists of lignin, cellulose and hemicellulose. Lignin is associated with carbohydrates in plant cell walls and is highly resistant to chemical degradation (McDonald et al. 2010). However, other researchers (Daniel et al. 2014; Harper and McNeill 2015) have reported that organic matter and fibre digestibility are directly related to the potentially digestible portion of NDF (pdNDF) in a feed. They defined the latter as the NDF fraction that disappeared after a long incubation period, leaving the indigestible component of NDF (iNDF) which is unavailable for microbial digestion. Hence, understanding the dynamics of fibre digestibility is key to minimizing the inhibitory effects of NDF on feed intake of bush-based feed. Furthermore, Harper and McNeill (2015) reported that iNDF is a useful index for predicting OM digestibility and its inclusion in feed evaluation models increases accuracy.

Other factors associated with the feeding value of high fibre diets is the enteric production of greenhouse gases (GHG) particularly methane. The production of methane by livestock and its impact on the environment is a worldwide concern because it contributes to global warming (Grossi et al. 2019; Aboagye and Beauchemin 2019). Methane (CH4) production is increased when feeds high in fibre are fed to ruminants (Goel and Makkar 2012). Considering that milled bush is also high in fibre, there is limited information on the methane production of these bush species. Therefore, the objective of the study was to evaluate the in situ NDF digestibility, in vitro OM digestibility and methane production of four predominant rangeland encroacher bushes in Namibia, namely *Senegalia mellifera*, *Dichrostachys cinerea*, *Terminalia sericea* and *Rhigozum trichotomum*.

## Materials and methods

### Harvesting and processing of the browse materials

The milled bush materials of encroacher bush species used in this study was of the predominant encroacher species, namely *Senegalia mellifera*,* Dichrostachys cinerea*, *T*. *sericea* and *R*. *trichotomum*. Samples of leaves and twigs of 20 mm or less in stem diameter from the selected encroacher bush species were harvested with a pruning scissor. Samples were collected in August 2018 (late dry season) and December 2018 (early rainy season). The fresh biomass was milled using a hammer mill (Retsch Mable mill; Retsch GmbH) to a particle size of 10 mm and air dried under shade until constant weight, stored in tightly closed plastic bottles until required for laboratory analysis. Table 1 shows the nutrient composition of the bush materials.

**Table 1:** The chemical composition (g/kg DM) of four bush encroacher species in different seasons

Bush species	Season	Components*					
		DM	OM	CP	NDFom	ADFom	ADLom
*D*. *cinerea*	Late DS	939.6	907.7	66.5	734.3	578.6	163.4
	Early RS	954.3	916.5	88.8	689.3	569.1	184.4
*R*. *trichotomum*	Late DS	940.5	900.3	74.9	594.3	463.2	137.5
	Early RS	952.7	917.3	74.3	641.6	505.9	153.1
*S*. *mellifera*	Late DS	934.0	883.9	82.1	667.7	518.8	144.1
	Early RS	957.0	906.1	111.8	647.4	511.6	144.1
*T*. *sericea*	Late DS	924.0	856.9	46.9	617.6	504.3	148.7
	Early RS	952.5	890.5	47.9	620.2	522.2	168.0

*DM, dry matter; *OM*, Organic matter; *CP*, Crude protein; *NDFom*, ash-free neutral detergent fiber; *ADFom*, ash-free acid detergent fiber; *ADLom*, ash-free acid detergent lignin; *DS*, dry season (August – November); *RS*, Rainy season (December – March)

### In situ neutral detergent fibre digestibility

Ten replicates for each bush species with two subsamples (duplicates) were placed in the rumen of each cow giving a total of 20 nylon bags per species per cow per season. The indigestible neutral detergent fibre (iNDF) and digestible neutral detergent fibre (dNDF) were determined by the in situ method, after incubating nylon bags with bush samples in the rumen of two non-lactating Finnish Ayrshire cows fitted with rumen cannula for a period of 12 days (Ahvenjärvi et al. 2000; Nousiainen et al. 2003). The two cows used in the determination were fed with grass silage and minerals. The silage contained 480 g/kg of dry matter (DM), and 604, 128, 122 and 70 g/kg DM of NDF, iNDF, crude protein and ash, respectively. The nylon bags (Sefar Petex^®^, Switzerland; external dimensions 60 × 120 mm) with small pore size of 17 μm were used to minimize particle inflow and outflow, but still ensuring sufficient microbial activities inside the bags (Jančík et al. 2008).

A bush sample weight of 4 g was weighed into each nylon bag and incubated in duplicates in each cow. The bags were collected from the rumen after the incubation period of 12 days, first rinsed by hand and then in a washing machine with cold water for 1 h. Following the procedure described by Ahvenjärvi et al. (2000) and Nousiainen et al. (2003), the same bags with samples were dried at 100 °C in a forced-air oven for 48 h. These samples were further weighed and boiled in neutral detergent solution for 1 h to remove microbial and endogenous matter. The samples were again rinsed in cold water by hand and then dried at 100 °C for 48 h and weighed. The iNDF concentration (g/kg DM) was expressed as NDF residue after rumen incubation. The NDF concentration was determined in the presence of sodium sulfite (Van Soest et al. 1991) using a Tecator Fibertec system (1020 hot extractor and 1021 cold extractor; Foss). Digestible NDF (dNDF) (g/kg DM) was calculated as NDF-iNDF.

### In vitro organic matter digestibility

The experimental design for the in vitro organic matter digestibility was a complete random design with ten (10) replicates for each bush species per season. The in vitro organic matter digestibility (OMD) and the content of digestible organic matter in dry matter (D-value) of bush samples were based on the two-stage pepsin + cellulase solubility technique as described by Nousiainen et al. (2003). Approximately 200 mg of bush samples ground to pass through a 1 mm sieve (Retsch Mable mill; Retsch GmbH) were weighed into glass beakers. For the first step, samples were pre-treated with 20 ml pepsin (Merck KGaA, Germany) in 0.1 N HCl solutions and then shaken before incubating at 40 °C for 24 h, using a laboratory conventional oven. After incubation, the same sample and solution were heated at 100 °C for 10 min. The samples were washed with cold water and filtered through sintered glass crucible, porosity No.3. For the second step, 30 ml of cellulase (Cellulase “ONOZUKA” R-10; Yakult Pharmaceutical IND.CO, LTD) in an acetate-acetic acid buffer solution was added to the sintered glass crucibles and placed in beakers and incubated at 40 °C for 48 h. At the end of incubation, the samples were washed with hot distilled water, rinsed with acetone, dried at 100 °C and then incinerated in a muffle furnace at 500 °C for 3 h.

The in vitro digestibility values were calculated using the formulas of Huhtanen et al. (2006):

$${\rm{OMD = (OMi - OMf)/OMi}}$$

$${\rm{D-value = (OMi - OMf)/DMi}}$$

Where, OMi: initial amount (g) of organic matter incubated (obtained by subtracting the ash content from the total dried initial sample weight (100%)); OMf: final amount (indigestible residue) of organic matter after incubation process (g); DMi: initial amount (g) of dry matter incubated.

### In vitro methane determination

The experimental design for the in vitro methane determination was a complete random design with three (3) replicates for each bush species in each of the two (2) seasons. Methane (CH4) gas production was determined by in vitro method using the Gas Endeavour Automatic Gas Flow Measuring System (Bioprocess Control, Lund, Sweden), after incubating bush samples with rumen liquor collected from two Finnish Ayrshire dry cows fitted with rumen cannulas. According to Yáňez-Ruiz et al. (2016), the diet of donor animals should resemble that of incubated feeds in in vitro systems. In this experiment, donor cows were fed grass silage, supplemented with minerals, which is a high forage diet, exclusive of energy concentrates, to ensure efficient fibre digestion for incubated fodder samples. Rumen digesta were collected at four different sites in the rumen into plastic bottles and the sealed bottles were placed in warm water bath at 40 °C.

Rumen digesta was filtered through a 250 μm sieve under nitrogen flush in the laboratory. The buffer was prepared following the method of McDougall (1948). The prepared buffer was mixed with rumen inoculum in a ratio of 2:1 to make 400 ml of incubation media per incubation bottle. About 6 g of dried sample milled through a 1 mm sieve were added to each incubation bottle of 500 ml in size, then incubated at 39 °C for 24 h in triplicates, and one blank (without feed) to correct for CH4 production of residual organic matter in the inoculum. In addition, duplicates of grass silage (1st harvest in Helsinki, Finland; CP 146, NDF 515, iNDF 63.6, total fat 25.6, ash 74.7 and D value 693 g/kg DM) served as control samples. Incubation bottles and lines were flushed with N_2_ at the beginning of the incubation to obtain anaerobiosis.

The Gas Endeavour Automatic Gas Flow Measuring System consisted of a sample incubation unit, a gas absorption unit with alternating clockwise and counterclockwise stick stirring at 39 °C in a water bath, a gas absorption unit containing 3 M NaOH to capture CO_2_, and a gas measuring unit with 9 ml flow cells connected to web-based software running on embedded server. After 9 ml of gas accumulated into the flow cell spoon, it was deliberated, and the number of spoon openings were continuously recorded.

An integrated embedded data acquisition system was used to record, display and analyse the results. The bottle contents were dried at 103 °C to determine undigested residue after the incubations. Methane gas production results were calculated at 0 °C, 1 standard atmosphere (atm) and dry gas.

### Statistical analysis

The data for in situ neutral detergent fibre digestibility were analysed as a randomized complete block design (RCBD) with subsampling (Lentner and Bishop 1993) using the General Linear Model (GLM) procedure (SAS Institute Inc 2009). The cows were the blocks and the other effects in the model were species, season and species x season interactions. The duplicate nylon bags for each treatment combination were the subsamples. Data on digestible neutral detergent fibre (dNDF) was transformed using logarithms to ensure it had an approximate normal distribution before analysis. The following statistical model was used:

$${{\rm{Y}}_{{\rm{ijkl}}}}{\rm{ = \mu + }}{{\rm{B}}_{\rm{i}}}{\rm{ + }}{{\rm{S}}_{\rm{j}}}{\rm{ + }}{\left( {{\rm{BS}}} \right)_{{\rm{ij}}}}{\rm{ + }}{{\rm{C}}_{\rm{k}}}{\rm{ + }}{{\rm{e}}_{{\rm{ijk}}}}{\rm{ + }}{{\rm{\delta }}_{{\rm{ijkl}}}}$$

Where:

Y_ijkl_ = dependent variable (in vitro OMD, iNDF, dNDF, etc.);

µ = overall mean;

B_i_ = effect of browse species (i = 1, 2, 3, 4);

S_j_ = effect of season (j = 1, 2);

(BS)_ij_ = interaction effect of browse species and season;

C_k_ = effect of cow (k = 1, 2);

e_ijk_ = random component explaining variation among experimental units (EU) on the same treatment within blocks;

δ_ijkl_ = random variation within the EU (among the subsamples).

Data on in vitro organic matter digestibility and methane production was analysed as a random complete design (RCD) using the General Linear Model (GLM) procedure (SAS Institute Inc 2009). Methane data was transformed using the fourth root to make it approximately normal, prior to analysis; the least squares means were then back transformed to the original scale.

For the statistical analysis, seasons were defined as late dry season [Late DS] (August –November); early rainy season [Early RS] (December – March). Means were separated using the Tukeys’ Studentised range test. Differences among means with *P* < 0.05 were considered significant.

## Results

### In situ neutral detergent fibre digestibility

The in situ NDF digestibility parameters of the four encroacher species are presented in Table 2. Species, season and species x season interaction influenced (*P* < 0.0001) the in situ NDF digestibility. The iNDF_DM for *D*. *cinerea* and *S*. *mellifera* decreased (*P* < 0.0001) as season progressed from late dry to early rainy season, while that of *R. trichotomum* and *T*. *sericea* increased. *Terminalia sericea* and *S*. *mellifera* had lower iNDF_DM and iNDF_NDF compared to the other two species.

The digestible NDF (dNDF) for all species decreased (*P* < 0.0001) from dry season to early rainy season, except for *S*. *mellifera*. Lower (*P* < 0.001) digestible NDF (dNDF) values were observed for *D*. *cinerea* and *R*. *trichotomum* compared to the other species.

### In vitro organic matter digestibility

Species and species x season interaction affected (*P* < 0.0001) the in vitro OMD and D-value (Table 2) of the four browse species. Except for *S. mellifera*, the in vitro OMD and D-value for other species decreased (*P* < 0.0001) from dry season to early rainy season. Lower (*P* < 0.001) in vitro OMD and D-value were observed for *D. cinerea* in both seasons compared to other species. The D-value also followed the same pattern as the in vitro OMD in both seasons.

**Table 2:** In situ NDF and in vitro organic matter digestibility of four encroacher bush species collected in two different seasons

Components*							
**Bush species**	**Season**	**dNDF_DM** (g/kg DM)	**dNDF_NDF** (g/kg NDF)	**iNDF_DM** (g/kg DM)	**iNDF_NDF** (g/kg NDF)	In vitro **OMD** (g/kg OM)	**D value** (g/kg DM)
*D*. *cinerea*	Late DS	102.5^c^± 1.8	139.6^c^ ± 2.6	631.0^a^ ± 2.7	859.1^c^ ± 2.6	313.5^d^ ± 3.1	303.7 ^e^ ± 2.8
	Early RS	81.6^d^± 1.8	118.6^d^ ± 2.6	605.5^b^ ± 2.7	879.1^b^ ± 2.6	294.7^e^ ± 3.1	283.5^f^ ± 2.8
*R*. *trichotomum*	Late DS	74.9^e^± 1.8	126.2^d^ ± 2.6	518.9^d^ ± 2.8	872.8^b^ ± 2.6	424.0^a^ ± 3.1	407.5^a^ ± 2.8
	Early RS	53.5^f^± 2.0	83.4^e^ ± 2.8	587.2^c^ ± 3.0	915.5^a^ ± 2.8	383.8^c^ ± 3.2	370.5^c^ ± 2.9
*S*. *mellifera*	Late DS	170.4^a^± 1.8	255.4^b^ ± 2.6	496.4^e^ ± 2.7	743.5^e^ ± 2.6	411.0^b^ ± 3.1	388.1^b^ ± 2.8
	Early RS	169.0^a^± 1.8	261.3^a^ ± 2.6	477.8^f^ ± 2.7	737.7^ef^ ± 2.6	424.4^a^ ± 3.1	402.8^a^ ± 2.8
*T*. *sericea*	Late DS	163.2^a^± 1.8	264.5^a^ ± 2.6	453.7^h^ ± 2.7	735.0^f^ ± 2.6	405.5^b^ ± 3.1	378.0^c^ ± 2.8
	Early RS	151.8^b^± 2.0	245.4^b^ ± 2.8	466.7^g^ ± 3.0	754.1^d^ ± 2.8	384.1^c^ ± 3.2	360.2^d^ ± 2.9
P-value	Species	< 0.0001	< 0.0001	< 0.0001	< 0.0001	0.02	0.02
	Season	< 0.0001	< 0.0001	< 0.0001	< 0.0001	0.23	0.26
	Species x Season	< 0.0001	< 0.0001	< 0.0001	< 0.001	< 0.0001	< 0.0001
R-square		0.95	0.96	0.94	0.96	0.94	0.94

*DM, dry matter; *NDF*, neutral detergent fibre; *iNDF_DM*, indigestible neutral detergent fibre in DM; *iNDF_NDF*, indigestible neutral detergent fibre in NDF; *dNDF_NDF*, digestible neutral detergent fibre in NDF; *OMD*, organic matter digestibility; *OM*, organic matter; *D-value*, digestible OM in DM; Values presented as Mean ± Standard Error (SE); ^a −h^Means with different superscripts within a column differ significantly **(***P* < 0.05**)**; *DS*, dry season (August – November); *RS*, Rainy season (December – March)

### In vitro methane gas production

Methane gas production from in vitro fermentation was influenced by season (*P* = 0.0004) (Table 3). The least squares means of methane gas production of digested DM at 24 h were 132.4 ± 6.6 mL/g for the late dry season and 92.7 ± 6.6 mL/g for the early rainy season.

**Table 3:** Methane gas production at 24 h (mL/g) of digested DM from four encroacher bush species collected in two different seasons

Bush species	Season	Methane (24 h)
*D. cinereal*	Late DS	144.9^a^
	Early RS	91.5^b^
*R. trichotomum*	Late DS	108.7^ab^
	Early RS	92.3^b^
*S*. *mellifera*	Late DS	147.6^a^
	Early RS	94.8^b^
*T*. *sericea*	Late DS	128.5^ab^
	Early RS	92.0^b^
SEM		13.7
P-value	Species	0.415
	Season	0.003
	Species x Season	0.521

^**ab**^Means with different superscripts within a column differ significantly **(***P* < 0.05**);** *SEM*, Standard error of means; *DS*, dry season (August – November); *RS*, Rainy season (December – March)

## Discussion

### In situ neutral detergent fibre digestibility

Climate change affects the growth of rangeland herbaceous and woody vegetation components. The rangeland woody vegetation is a major contributor to animal feed for both livestock and game in many countries around the world as the herbaceous vegetation fails to grow due to limited rainfall. The nutritional characterisation of rangeland woody vegetation is therefore important. The indigestible NDF (iNDF) represents the fraction of feed that is not digested and hence it is a nutritionally critical input factor in feed evaluation systems when estimating the potential NDF digestibility (Ellis et al. 1999). Phenolics mostly in leaves and lignin in stems have been widely reported as major factors limiting digestibility of NDF (Woodward and Reed 1989; Soufizadeh et al. 2018).

Lignin restricts digestion of all insoluble fibre fractions and by its complex association with other cell wall components. Lignin constitutes the main barrier to fibre digestion (Traxler et al. 1998; Adesogan et al. 2019) through reduction of enzyme access to cellulose and xylan and adsorption of enzymes, hence reducing rates of hydrolysis of structural polysaccharides (Bansal et al. 2009). The environment, plant maturity and genetics influences the rate and extent of potential NDF digestibility (da Cruz et al. 2021).

Harper and McNeill (2015) proposed that the iNDF fraction of forage is attributable to the cross-linking between cell wall lignin and hemicellulose. However, the concentration of lignin is much higher in stems than in leaves, which limits the digestion of the NDF fraction in the high-fiber forage. Hence, the inclusion of a high proportion of branches of 20 mm thickness in the samples of bushes used in this study could be one of the contributing factors to the general high iNDF observed. The implication is that forage digestibility of milled bush fodder from the species considered would be low, which reduces intake, particularly during the late rainy season.

It appears that the season by species interaction for iNDF content observed in the bush species could be linked to the nature of the lignin-carbohydrate complex existing in different species (Harper and McNeill 2015) in combination with their varying concentration of phenolic contents during different seasons. The differences in NDF digestibility also partly reflect the differential species responses to environmental cues as a function of the agro-ecological zones in which they are found.

The encroacher species were harvested from different agro-ecological zones which differed in the onset of the rainy season, hence contributing to the significant species x season interaction effects on iNDF and OM digestibility. Most encroacher species in our study had elevated levels of total phenolics in the early rainy season compared to the dry season (Shiningavamwe 2021), which adversely affected components of NDF digestibility. Similarly, Scogings et al. (2004) reported elevated condensed tannin and total phenols in the growing rainy season compared to the dormant winter season. This was interpreted as a defensive mechanism of shrubs against browsers, which minimises destruction of apical buds (Rhoades 1979).

### In vitro organic matter digestibility

Digestibility is a major determinant of nutritive value of a feed (McDonald et al. 2010; Dambe et al. 2015) and studies on the digestibility of bush fodders are very important as they allow the estimation of nutrients available to the animal (Sanon et al. 2007). Organic matter digestibility is greatly influenced by indigestible NDF (Nousiainen et al. 2004) which is of importance in prediction of nutritive value of forages (Nousiainen et al. 2003). The in vitro OM digestibility was negatively correlated to the indigestible NDF content. The close association of cellulose and hemicellulose with lignin and hydroxycinnamic acids such as ferulic acid in the plant cell wall poses a huge barrier to the utilisation of forages and byproducts (Adesogan et al. 2019). Results of a study conducted on grasses by Jančík et al. (2010) indicated that the OM digestibility of grasses was attributed to differing maturing rates, which in turn were linked to the proportion of cell wall concentrations of phenolic acids and lignin.

Jung and Allen (1995) reported that leaf morphology of plants changes with seasons and within plant species. Lignin content of browse leaves varies with season, increasing with warmer temperatures (summer) and decreasing during lower temperatures (winter) due to limited light for plant development (Tjelele 2007; Ravhuhali et al. 2022). The close association of lignin with plant cell walls could therefore explain the reduction in in vitro OM digestibility as the season progresses from winter to summer. Sanon et al. (2007) and Matlebyane et al. (2009) reported that most tropical bushes have a high proportion of protein bound to fibre, which could limit the supply of nitrogen from the bush feed to rumen microbes in order to obtain maximal rate of digestion of the fibre.

Apart from low CP and high fibre fractions, Reed (1995) reported that condensed tannins present in most bush materials, could also be responsible for decreasing OM digestibility. The negative effect of tannins on rumen fermentation and OM digestion could be related to the formation of tannin carbohydrate and tannin protein complexes that are less degradable or toxic to rumen microbes (Gemeda and Hassen 2015). Although phenolics were assayed in all the species, their concentration was moderate (50–100 g/kg DM), except for *T. sericea* in the early rainy season (Shiningavamwe 2021). Hence in this study, the low OM digestibility most likely can be attributed to the lignin component, due to the inclusion of stem materials ≤ 20 mm thick. The implications of the high iNDF and low OM digestibility of encroacher bush material is that ration formulation should be aimed at enhancing fibre digestibility to improve intake.

Given the low organic matter digestibility of bush feed, it may be desirable to use alkali or enzyme treatment to improve its utilization in ruminant diets (Adesogan et al. 2019). Forages generally have low crude protein and phosphorus levels during the dry season, but these increase during the growing (summer) season (Scogings et al. 2004). Therefore, the provision of protein supplements may improve rumen microbial activity and hence fibre digestibility, contributing to better utilization of rangelands.

### In vitro methane production

Reducing enteric methane from ruminants has become a focus of animal nutrition, particularly in countries where agriculture is a major economic enterprise, as it causes significant energy losses in the rumen during the utilisation of feed energy (Theart et al. 2015; Cuervo et al. 2025). Methane gas production from encroacher bushes in this study were higher than those reported by Theart et al. (2015), in some Kalahari browse species and by Gemeda and Hassen (2015) in some tropical browse plants, which ranged from 0.10 to 64 ml/g DM. The large variation in methane gas production observed in other reports, even for the same species, could be associated with the assay method used, the species investigated, the age of the plant, the proportion of leaves to twigs, and the season of harvest (Makkar 2003). On the other hand, de Klerk (2016) observed increased methane production from diets with increased fibre contents. This could be also another compounding factor to the high methane production observed in this study given the fact that the browse products used contained high proportions of stem parts, hence high fibre, especially in the dry season when bushes had few or no leaves. Other studies observed seasonal changes in water-soluble carbohydrates and short chain fatty acids (SCFA) in grass silages which have been suggested to contribute to changes in methane production (Weiby et al. 2022). In this study, the WSC and SCFA were not assessed, however, the decreased lignin content during the cold months (May – September) could explain the increased digestibility which is associated with increased methane production in winter. Increased digestibility has been linked to increased CH_4_ yield per unit of gross energy or DM intake (Ramin and Huhtanen 2013), which is collaborated by our results (Tables 2 and 3).

The reduction of methane production from late dry to early rainy season could also be related to their increased phenolic and tannin concentrations observed as the season progressed from late dry to early rainy season as reported by Shiningavamwe (2021). This agrees with the observations by Gemeda and Hassen (2015) and Makkar (2003) that browse plants with higher phenolic and tannin contents generally produced lower methane gas regardless of their CP, NDF, ADF and lignin contents. Furthermore, there was a reduction in dietary fiber digestibility, which could possibly reduce methane production because H_2_ is released during fibre fermentation, and it acts as a substrate for methanogenesis (Moss et al. 2000; Tavendale et al. 2005; Cuervo et al. 2025).

Except for *S. mellifera*, the higher the in vitro OM digestibility and the D-value (Table 2), the higher the methane production (Table 3). This apparent contradiction between forage digestibility and methane production has also been reported for grass silages (Holtshausen et al. 2012; Weiby et al. 2022) and fresh pastures (Jonker et al. 2016). This presents a dilemma for rangeland improvement so as to enhance livestock productivity because selecting forages with greater digestibility (and hence better average daily gain) would lead to an apparent increase in methane production and hence increase in emissions intensity (CH4/kg average daily gain). Species like *S. mellifera* that have unique attributes of increased in vitro OM digestibility during the early rainy season, while the methane production remains low, may have to be considered more favourably in rangeland restoration because of the desirable environmental outcome.

### Potential implications of findings for rangeland management and livestock production

The study provides evidence that it is possible to utilize biomass from the four encroacher bush species as fodder for livestock while simultaneously contributing to the control of bush encroachment. Bush control has been declared by the Namibian government as a priority in its fifth National Development Plan (NDP5), to address the land degradation problem (Wilkie and David 2020). Thinning out encroacher bushes can mitigate their negative environmental impact on water availability for grasses (Boshoff 2024), and subsequently increase grass biomass production which, in the long run, would enhance rangeland carrying capacity (Nghikembua et al. 2021). It is envisaged that restoration of degraded land and improved livestock productivity will have a positive effect on ecosystem services and, by inference, increase farming income streams and the well-being of the farming community (Sebitloane et al. 2020; Kabeta et al. 2020).

The study findings indicated that using milled bush as a sole feed for ruminants may be limited by their relatively low in vitro OMD and in situ neutral detergent fibre digestibility. Enhancement of bush-based feed will require enzyme or chemical treatments to degrade the lignin and enhance fibre digestibility.

Seasonal variation effects on the digestibility and methane production will also inform the harvesting of bushes at a time when digestibility and methane production is expected to be optimal to support quality fodder production. The differential NDF and OM digestibility among encroacher species by season implies that maintaining diversity of encroacher species is advantageous for ecosystem functionality, rangeland restoration and livestock production as it would enhance security of feed supplies throughout the year.

The effects of climate change are already evident in Southern Africa as exemplified by the recurring droughts in Namibia. Hence, utilization of encroacher bushes that are deep-rooted with ability to access water at greater depths gives them an edge over shallow-rooted grasses, by contributing to climate change adaptation as a fodder resource for livestock. Therefore, the findings of the study contribute to the understanding of the nutritional value of bush biomass, as the farmers’ focus shifts towards bush-based feed as an alternative natural feed source and emerging prominent solution (Boshoff 2024). This approach allows for utilization of available resources to sustain livestock productivity during periods of feed scarcity. Producing bush-based feed can also potentially reduce importation of livestock feed in Namibia which is currently ranking as the eighth most imported product amounting to US$231 million (Nkonya et al. 2016). The limitations of this study are the small sample size, the lack of sampling across different years and the high degree of temporal-spatial variability in the nutrient content of the encroacher species.

## Conclusion

All species considered in this study were relatively low in vitro OMD and in situ neutral detergent fibre digestibility which constrains them as a sole feed resource for ruminants and may require chemical or enzyme treatments and supplementation to improve their utility. Seasonal variability in fibre and phenolics may affect digestibility and methane production of the four encroacher bush species. Dietary manipulation is required to improve organic matter and neutral detergent digestibility while reducing the methane gas production from the species studied.

## Data Availability

The datasets generated for the current study are available from the corresponding author on reasonable request.
